# Characterization of *Rhizobium grahamii* extrachromosomal replicons and their transfer among rhizobia

**DOI:** 10.1186/1471-2180-14-6

**Published:** 2014-01-08

**Authors:** María Julia Althabegoiti, Ernesto Ormeño-Orrillo, Luis Lozano, Gonzalo Torres Tejerizo, Marco Antonio Rogel, Jaime Mora, Esperanza Martínez-Romero

**Affiliations:** 1 Programa de Ecología Genómica, Centro de Ciencias Genómicas, Universidad Nacional Autónoma de México, Av. Universidad s/n, Col. Chamilpa, C.P. 62210, Cuernavaca, Morelos, Mexico; 2 Programa de Genómica Evolutiva, Centro de Ciencias Genómicas, Universidad Nacional Autónoma de México, Av. Universidad s/n, Col. Chamilpa, C.P. 62210, Cuernavaca, Morelos, Mexico; 3 Programa de Ingeniería Genómica, Centro de Ciencias Genómicas, Universidad Nacional Autónoma de México, Av. Universidad s/n, Col. Chamilpa, C.P. 62210, Cuernavaca, Morelos, Mexico; 4 Programa de Genómica Funcional de Procariotes, Centro de Ciencias Genómicas, Universidad Nacional Autónoma de México, Av. Universidad s/n, Col. Chamilpa, C.P. 62210, Cuernavaca, Morelos, Mexico

**Keywords:** Genome sequence, Quorum sensing, Symbiotic plasmid, Conjugative transfer

## Abstract

**Background:**

*Rhizobium grahamii* belongs to a new phylogenetic group of rhizobia together with *Rhizobium mesoamericanum* and other species. *R. grahamii* has a broad-host-range that includes *Leucaena leucocephala* and *Phaseolus vulgaris*, although it is a poor competitor for *P. vulgaris* nodulation in the presence of *Rhizobium etli* or *Rhizobium phaseoli* strains. This work analyzed the genome sequence and transfer properties of *R. grahamii* plasmids.

**Results:**

Genome sequence was obtained from *R. grahamii* CCGE502 type strain isolated from *Dalea leporina* in Mexico. The CCGE502 genome comprises one chromosome and two extrachromosomal replicons (ERs), pRgrCCGE502a and pRgrCCGE502b. Additionally, a plasmid integrated in the CCGE502 chromosome was found. The genomic comparison of ERs from this group showed that gene content is more variable than average nucleotide identity (ANI). Well conserved *nod* and *nif* genes were found in *R. grahamii* and *R. mesoamericanum* with some differences. *R. phaseoli* Ch24-10 genes expressed in bacterial cells in roots were found to be conserved in pRgrCCGE502b. Regarding conjugative transfer we were unable to transfer the *R. grahamii* CCGE502 symbiotic plasmid and its megaplasmid to other rhizobial hosts but we could transfer the symbiotic plasmid to *Agrobacterium tumefaciens* with transfer dependent on homoserine lactones.

**Conclusion:**

Variable degrees of nucleotide identity and gene content conservation were found among the different *R. grahamii* CCGE502 replicons in comparison to *R. mesoamericanum* genomes. The extrachromosomal replicons from *R. grahamii* were more similar to those found in phylogenetically related *Rhizobium* species. However, limited similarities of *R. grahamii* CCGE502 symbiotic plasmid and megaplasmid were observed in other more distant *Rhizobium* species. The set of conserved genes in *R. grahamii* comprises some of those that are highly expressed in *R. phaseoli* on plant roots, suggesting that they play an important role in root colonization.

## Background

A large proportion of *Rhizobium*, *Sinorhizobium* and *Agrobacterium* genomes is located in extrachromosomal replicons (ERs) [1]. ERs play adaptive roles in soil bacteria [1,2] and are enriched in particular classes of genes involved in pathogenesis, symbiosis, metabolism and antibiotic resistance. Two types of ERs have been recognized, chromids [3] and plasmids. The term chromid has been recently proposed to refer to extrachromosomal elements that carry “essential” genes and have similar G + C content and codon usage as chromosomes [3]. Nodulation and nitrogen fixation genes are located on symbiotic plasmids (pSyms) in *Rhizobium, Sinorhizobium, Burkholderia* and in some *Mesorhizobium* species [1,4] but in some cases these genes may reside in chromids. pSyms determine the symbiotic capacities in rhizobia and may be transferred among bacteria. The term symbiovar refers to host specificity. A single symbiovar may be present in different rhizobial species while a single species may exhibit different symbiovars [5]. Well conserved pSyms have been found respectively in rhizobia nodulating *Phaseolus vulgaris* corresponding to symbiovars (sv) tropici or phaseoli [6,7], and we wondered if conserved pSyms are a rule or an exception in rhizobia [8]. An “acaciella” symbiotic plasmid seems to be contained in the related *Ensifer* (also named *Sinorhizobium*) species, *E. mexicanum* and *E. chiapanecum*[9]. Symbiovar mimosae is found in the related species *Rhizobium etli* and *Rhizobium phaseoli* and symbiovar meliloti is the most widespread found in several *Ensifer* or *Mesorhizobium* species [5].

A novel phylogenetic group in rhizobia is now recognized for *Rhizobium grahamii, Rhizobium mesoamericanum*[10], *Rhizobium endophyticum*[11], *Rhizobium* sp. OR191 [12], *Rhizobium* sp. LPU83 [13], *Rhizobium tibeticum*[14] and *Rhizobium* sp. CF122 [15]. *R. grahamii*, *R. mesoamericanum, Rhizobium* sp. OR191 and *Rhizobium* sp. LPU83 are broad host range bacteria. They are capable of forming nodules on *P. vulgaris* although they are not fully efficient or competitive. *R. endophyticum* is non-symbiotic as it lacks a symbiotic plasmid [11]. *R. grahamii* and *R. mesoamericanum* are closely related species. *R. grahamii* strains have been isolated from nodules of *Dalea leporina, Leucaena leucocephala* and from *Clitoria ternatea* growing naturally as weeds in agricultural bean fields in central Mexico [16]; or from *P. vulgaris* nodules. *R. mesoamericanum* strains have been isolated from *Mimosa pudica* in Costa Rica, French Guiana and New Caledonia [17-19] and from *P. vulgaris* nodules in Los Tuxtlas rain forest in Mexico [10]. Seemingly, *R. mesoamericanum* strains were introduced to New Caledonia together with their mimosa hosts [18], maybe on seeds as described before for other rhizobia [20].

Genome sequences are available for *R. grahamii*, *R. mesoamericanum*[10,21] and *Rhizobium* sp. CF122 [15]. Whole genome comparison of related species would provide clues on the divergence mechanisms involved in speciation. Numerical estimates such as average nucleotide identity (ANI) and genome conservation estimates have been found useful to globally compare genomes [22], and we use them here. In this work we present 1) an improved version of the *R. grahamii* CCGE502 genome, 2) a genomic comparison of ERs in related rhizobia, 3) evidence of the natural integration of an ER in the *R. grahamii* CCGE502 chromosome, and 4) an evaluation of the conjugative transfer ability of the *R. grahamii* CCGE502 symbiotic plasmid and megaplasmid to other *Rhizobium* species.

## Methods

### Bacterial strains and growth conditions

The bacterial strains and plasmids used in this work are described in Table 1. *Rhizobium* and *Agrobacterium tumefaciens* strains were grown at 30°C on PY medium [23]. *Escherichia coli* cells were grown on LB medium [24] at 37°C. When required, antibiotics were added at the following concentrations (in μg ml^-1^): nalidixic acid (Nal) 20, spectinomycin (Sp) 75, kanamycin (Km) 15, neomycin (Nm) 60, rifampicin (Rif) 100, streptomycin (Sm) 50, gentamicin (Gm) 30.

**Table 1:** Bacterial strains, plasmids and primers

**Strain**	**Relevant characteristics**	**Source**
**Rhizobia**		
*R. grahamii* CCGE502	Wild type strain	[10]
*R. mesoamericanum* CCGE501	Wild type strain	[10]
*R. mesoamericanum* CCGE501-1	mini-Tn5 Sm^R^/Sp^R^	This work
*R. grahamii* CCGE502a:GFP	CCGE502 carrying a Gm: GFP cassette at pRgrCCGE502a	This work
*R. grahamii* CCGE502b:Km	CCGE502 carrying pK18mob:*sacB* at	This work
*R. grahamii* CCGE502*ΔtraI*	CCGE502 carrying a deletion of *traI*.	This work
*R. grahamii* CCGE502*ΔtraI::nodC*	CCGE502Δ*traI* with pG18mob2 inserted at *nodC*	This work
*R. etli* CFN2001	CFN42 derivative (pRetCFN42a^-^pRetCFN42d^-^)	[25]
*S. fredii* GR64-4	GR64 cured of pSfrGR64a and pSfGRr64b, Rif^R^	[26]
*S. meliloti* SmA818R	2011 cured of pSymA, Rif^R^	[27]
*R. phaseoli* Ch24-10	Tn5mob, Neo^R^	Rosenblueth, M, unpublished
*Rhizobium* sp. LPU83	Sm^R^	[27]
*R. endophyticum* CCGE2052	Endophyte of *P. vulgaris*	[11]
***Agrobacterium***		
GMI9023	C-58 cured of its native plasmids	[28]
GMI9023 (pRgrCCGE502a:GFP)	GMI9023 carrying pRgrCCGE502a with a Gm-GFP cassette	This work
GMI9023 (pRgrCCGE502b:Km)	GMI9023 carrying pRgrCCGE502b with a pK18mob:*sacB* insertion	This work
GMI9023 (pRgrCCGE502a:GFP, pRgrCCGE502b:Km)	GMI9023 carrying pRgrCCGE502a with a Gm: GFP cassette and pRgrCCGE502b with a pK18mob:*sacB* insertion	This work
GMI 9023 (Sp^R^)	GMI9023 with a mTn5SSgusA40	This work
GMI 9023(pRgrCCGE502a:GFP, pBBR1MCS2*::traI*)	GMI9023 carrying pRgrCCGE502a with a Gm-GFP cassette and pBBR1MCS2*::traI* overexpressing AHLs of *R. grahamii*	This work
***Escherichia coli***		
DH5α	Recipient for transformation, *sup*E44 *Δlac*U169 ϕ80l*acΔ*ZM15) *hsd*R17 *rec*A1 *end*A1 *gyr*A96 *thi*-1 *rel*A1	[29]
S17-1	*E. coli* 294 RP4-2-Tc::Mu-Km::Tn7 integrated into the chromosome	[30]
**Plasmids**		
pG18mob2	Cloning vector, Gm^R^	[31]
pK18mob:*sacB*	Cloning vector, Km^R^, *sacB*	[32]
pRK2013	ColE1 replicon, tra + de RK2, Km^R^	[33]
pCAM140	pUT/mini-Tn5 Sm^R^/Sp^R^	[34]
pMJAM01	A fragment of RGCCGE502_32801 cloned at *Sma*I in pK18mob:*sacB*	This work
pMJAM02	*Not*I cassette carrying Gm-GFP was cloned at pMJAM01	This work
pMJAM03	Fragment 1 of RGCCGE502_33766 cloned at *Sma*I in pK18mob:*sacB*	This work
pMJAM04	Fragment 2 of RGCCGE502_33766 cloned at *BamH*I-*Hind*III of pMJAM03	This work
pMJAM05	A *nodC* fragment cloned at *Sma*I pG18mob2	This work
pMJAM06	An intergenic region of pRgrCCGE502b cloned at *Sma*I in pK18mob:*sacB*	This work
pRgrCCGE502a-GFP	pRgrCCGE502a carrying a Gm-GFP cassette	This work
pRgrCCGE502b-Km	pRgrCCGE502b carrying pK18mob:*sacB*	This work
**Primers**	**Sequence 5′ 3′**	
M13 Fw	GTAAACGACGGCCAGT	
M13 Rv	GCGGATAACAATTTCACACAGG	
Fw_32801	GGGACACGCAGTCACCTTAG	This work
Rv_32801	GACGGGGAGCAAAGTTCAT	This work
Fw_ext_32801	GGACTATCTCGCCCTGACAA	This work
Rv_ext_32801	AAATCGCTGACAATCCCAAG	This work
Fw_33766_1	CGTTCCCGATCTGTTTATCTG	This work
Rv_33766_1	CACGGAGCTGATGATGGTT	This work
Fw_33766_2	AAAAA**GGATCC**CAGAAGGTCGGCGTAACAA	This work
Rv_33766_2	AAAAAA**AAGCTT**CCAGCCGTTCGATGAAGA	This work
Fw_ext_traI	GACGTGAATTTTCGCAGGA	This work
Rv_ext_traI	ATGGTGAAGGCGGGTTTAG	This work
Fw_nodC	ACACGGCTAATTGACATGGA	This work
Rv_nodC	CGAAAACCTGCCTTCAACA	This work
Fw_ext_nodB	CGCCAACCACACTATGACAC	This work
Rv_ext_nodC	GGGGACTTCTTGACTGTGGA	This work
Fw_28753	GATGCCTCCCTGTTCACTCT	This work
Rv_28753	CTGTAGGCTTCTCCGTCGAG	This work
Fw_ext_28753	GAGACGAGCCAGACGAAAAC	This work
Rv_ext_28753	ATCTGCAGCAGTCGAAGGAT	This work

Boldface letters indicate restriction enzyme recognition sites, used for cloning purposes.

### Bacterial matings

Conjugation of *E. coli* and *Rhizobium* was done biparentally, using *E. coli* S17-1 as the donor [30]. Transconjugants were selected with the appropriate antibiotics. Conjugation experiments were performed on PY plates at 30°C using overnight cultures. Donors and recipients were mixed in a 1:2 ratio and incubated overnight. The mixtures were serially diluted and plated on suitable selective media. To study conjugative transfer of the *R. grahamii* CCGE502 pSym, it was tagged (see below).

### PCR amplification and cloning

The oligonucleotides used in this study were purchased from Unidad de Síntesis Química, IBT-UNAM. PCR amplification was carried out with recombinant TaqDNA polymerase (Invitrogen) and PFU (Fermentas) as specified by the manufacturer. PCR products were purified with the High Pure PCR Purification Kit (Roche). Vectors were purified with the High Pure Plasmid Isolation Kit (Roche). T4 polynucleotide ligase was used as indicated by the manufacturer (Fermentas).

### Genetic manipulations

The symbiotic plasmid pRgrCCGE502a was tagged with a *Not*I-cassette carrying Gm and green fluorescence protein (Gm-GFP). GFP (gfpmut3*) protein was from plasmid pJBA28 [35] that harbors a *Not*I cassette with a *Ssp*I site. Gm was from pBSL142 [36] and cloned at *Ssp*I site. A fragment corresponding to RGCCGE502_32801 was amplified with PFU using Fw_32801 and Rv_32801 and cloned at the *Sma*I site of pK18mob:sacB obtaining pMJAM01. This plasmid was digested with *Not*I and the *Not*I- (Gm-GFP) cassette was ligated to obtain pMJAM02 in *E. coli* S17-1 that was mated with *R. grahamii* CCGE502. Transconjugants were plated on PY Gm and Nm, selecting single recombinants. These colonies were checked by PCR with Fw_ext_32801 and Rv_ext_32801, combined with internal primers of the vector. Once the orientation of the insert was verified, one colony was grown to stationary phase and plated on PY sucrose and Gm. Finally the colonies obtained were checked by PCR to confirm double recombination and were named *R. grahamii* CCGE502a:GFP.

A *traI* mutant was obtained by deletion of a 428 base pair (bp) internal fragment of this gene (locus tag RGCCGE502_33766, size 621 bp). Two fragments of the gene were amplified. The first 265-bp fragment was amplified with PFU using Fw_33766_1 and Rv_33766_1. The second 272-bp fragment was amplified with Fw_33766_2 and Rv_33766_2. Fragment 1 was cloned blunt-ended in *Sma*I-digested pK18mob:*sacB* to obtain pMJAM03; and fragment 2 was cloned as a *BamH*I-*Hind*III fragment in the same vector to obtain pMJAM04 where both fragments are in the same orientation. The final construction was transformed into *E. coli* S17-1. The procedure to obtain the mutant in *R. grahamii* CCGE502 was the same as described above: first, transconjugants were plated on PY Nm, to select single recombinants which were used to perform PCR reactions to detect deleted derivative strains. External primers to verify insertions were Fw_ext_traI and Rv_ext_traI. Fragments amplified with these primers were 1500 bp and 1001 bp for wild type strain and deleted mutants, respectively. The mutant was designated *R. grahamii* CCGE502*ΔtraI.*

The symbiotic plasmid pRgrCCGE502a carrying the *traI* deletion was tagged by insertion of pG18mob2 [31] in the *nodC* gene. An internal fragment of *nodC* was amplified with PFU, employing Fw_nodC and Rv_nodC and cloned blunt-end in the *SmaI* site of pG18mob2 to obtain pMJAM05. The construction was transformed into S17-1 and transferred by mating to *R. grahamii* CCGE502*ΔtraI*. Transconjugants were verified by PCR combining Fw_ext_nodB or Rv_ext_nodC and M13 primers. The resultant strain was designated *R. grahamii* CCGE502*ΔtraI::nodC.*

Megaplasmid pRgrCCGE502b was tagged by insertion of plasmid pK18mob:*sacB*[32] in an intergenic region between RGCCGE502_28748 and RGCCGE502_28753. A 692-bp fragment was amplified with PFU, Fw_28753 and Rv_28753 and cloned blunt-end in the *SmaI* site of pK18mob:*sacB* to obtain pMJAM06. The construction was transformed into S17-1 and transferred by mating to *R. grahamii* CCGE502. Recombinants were verified by PCR combining Fw_ext_28753 or Rv_ext_28753 and M13 primers. The strain was designated *R. grahamii* CCGE502b:Km.

### N-acyl-homoserine-lactone (AHL) detection

Autoinducers were detected by thin-layer chromatography (TLC) with the reporter plasmid pZLR4 [37] that contains the *traR* gene and *traG::lacZ* reporter fusion from pTiC58, independently cloned into the broad-host-range vector pBBR1MCS5 [38]. Extracts from *R. grahamii* CCGE502 and mutants were prepared from 5-ml cultures grown in PY medium. Briefly, cultures were extracted twice with equal volumes of ethyl acetate, bacteria were removed by centrifugation and supernatants evaporated to dryness. Residues from 5-ml cultures were dissolved in 50–100 μl of ethyl acetate.

### Eckhardt gel analysis

This was performed as described [39], with liquid early-exponential-phase cultures in horizontal gels with sodium dodecyl sulfate in agarose.

### Gap closure

Gap filling was done over the contigs of the sequence assembly AEYE01000000 [40]. Ten contigs corresponding to symbiotic plasmid pRgrCCGE502a and sixteen corresponding to megaplasmid pRgrCCGE502b were selected. A new assembly was done with Phrap assembler using the 454 pyrosequencing mate-paired reads and edited with Consed (23.0) program [41]. A total of 1920 contigs were obtained and compared with the scaffolds corresponding to pRgrCCGE502a and pRgrCCGE502b of the original assembly. Contigs that overlapped with the pRgrCCGE502a and pRgrCCGE502b scaffolds were selected and analyzed at their ends to obtain the sequence that protruded into the gap region. Those protruding sequences were edited manually to fill the scaffold gaps. The complete pRgrCCGE502a and pRgrCCGE02b sequences were aligned with Illumina reads using Consed to verify the coverage of the new molecules. In some cases these processes located small contigs (corresponding to IS or repetitive sequences) to close a gap. A final annotation of the new version AEYE02000000 was performed by the NCBI Prokaryotic Genomes Automatic Annotation Pipeline (PGAAP). The replicons gave an estimated genome size of 7,156 kbp.

### Sequence comparisons

Average nucleotide identity (ANI) between sequences and sequence conservation was calculated with JSpecies software [22].

### Phylogenetic inference

Multiple sequence alignments were performed with CLUSTAL_X version 1.83 [42] and manually checked with BioEdit [43]. Best-fit models of sequence evolution were selected for each gene with ProtTest 2.4, using the Akaike information criterion [44]. Maximum-likelihood phylogenies were constructed with PhyML 3 using subtree pruning and regrafting moves to improve tree topology [45]. Support for tree nodes was evaluated by the Shimodaira–Hasegawa-like approximate likelihood-ratio test implemented in PhyML.

## Results

The genome of *R. grahamii* CCGE502 consists of three circular replicons, one chromosome and two ERs: one megaplasmid and a symbiotic plasmid. The first draft sequence [40] consisted of ten contigs for the symbiotic plasmid pRgrCCGE502a and sixteen corresponding to the megaplasmid pRgrCCGE502b. The version described in this paper is version AEYE02000000.

### Chromosome

**Figure 1:**
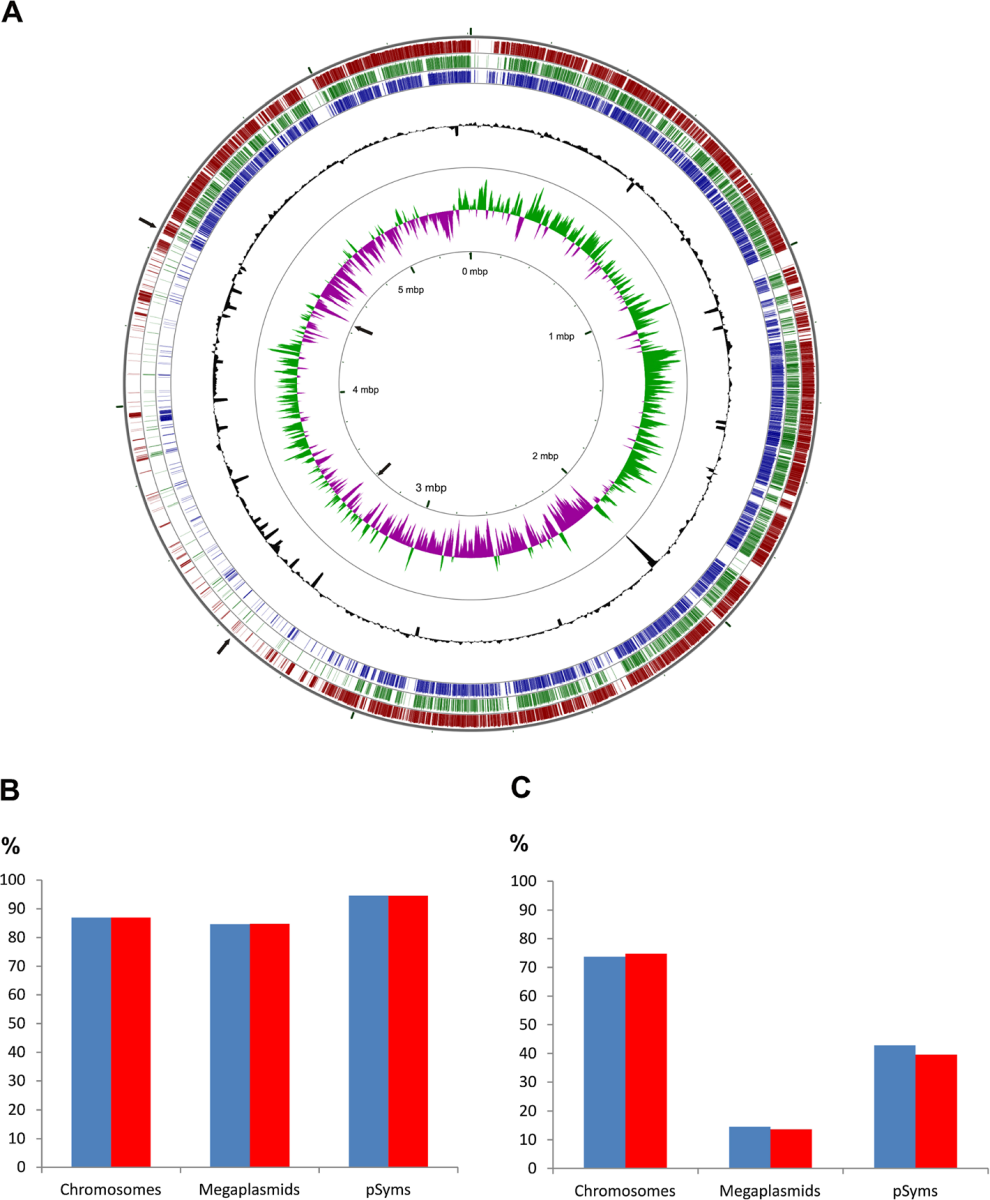
**Genomic comparison of *R. grahamii *and other rhizobia. A)** Chromosomal alignment of *R. grahamii* and other rhizobial chromosomes. Each replicon was split *in silico* in 10 kbp fragments and aligned by BlastN with *R. grahamii* CCGE502 chromosome as a reference (internal black circle with size labels). When 70% of identity in each fragment with the reference was found, a color line was used to indicate the conservation in the genomes. The colors used are: blue for *R. etli* CFN42, green for *R. tropici* CIAT 899 and red for *R. mesoamericanum* STM3625. The black circle with peaks represents the G + C content, and the outside internal circle the GC skew of *R. grahamii* CCGE502 chromosome. Black arrows indicate the location of the genomic island. **B)** ANI and **C)** conserved DNA values between replicons of *R. grahamii* CCGE502 and *R. mesoamericanum* CCGE501 (blue) or STM3625 (red).

The *ca.* 5,400-kbp chromosome of *R. grahamii* CCGE502 is the largest reported to date in *Rhizobium*. A genomic island of *ca.* 1,073 kbp that may have originated from the integration of a plasmid or an Integrative and Conjugative Element (ICE) [46] may account for its large size. Interestingly, this island has 57.1% G + C content, lower than the rest of the chromosome (59.7%) and the megaplasmid pRgrCCGE502b (59.1%), and more comparable to that of the symbiotic plasmid pRgrCCGE502a (57.4%). It is not similar to any known sequenced plasmid, and has a mosaic structure with genes resembling many different bacteria. It contains a *repABC* operon and a complete set of genes for a type IV secretion system. According to the latest classification of plasmid transfer systems proposed by Ding *et al.*[47] and based on the TraA relaxase and the TraG coupling protein phylogenies, the integrated replicon contains a type IVB rhizobial plasmid secretion system. However, the transfer mechanism of this new group still remains unclear. The chromosomal island encodes proteins related to chemotaxis, DNA metabolism and ABC transporters, among others. It is interesting to note that the location of the homologous genes in other bacteria is variable, they may be in plasmids or chromosomes. A BLASTN comparison of the *R. grahamii* CCGE502 chromosome with those of *R. mesoamericanum* STM3625, *Rhizobium tropici* CIAT 899 and *R. etli* CFN42 is shown in Figure 1A. Usually, the GC skew in bacterial chromosomes shows a bias toward G over the leading strand while the bias is to C on the lagging strand and indicates the origin of replication and the ending site [48]. In the *R. grahamii* chromosome the distinct GC skew indicates that the genomic island is a recent insertion. In order to validate that this integration is not an artifact of the assembly, we tagged the island by the insertion of a suicide vector containing a homologous region, to transfer the island to an *A. tumefaciens* free plasmid, but no transfer was detected. We also performed a Southern blot using a probe directed to the genomic island and hybridized a membrane of an Eckhardt gel. A signal was observed in the wells of the gel but not in the plasmids bands (not shown). Finally we did a PCR reaction employing primers outside and inside the genomic island and obtained a product of the expected size (not shown). Except for the genomic island, the *R. grahamii* chromosome is conserved with other rhizobial chromosomes (Figure 1A, Additional file 1: Table S1).

### Megaplasmid pRgrCCGE502b

The megaplasmid of *R. grahamii* CCGE502 appears to conform to the definition of a chromid; it had a similar G + C content as the chromosome (59.1% and 59.7% respectively), a plasmid-type maintenance and replication systems (*repABC)* and a group of genes present in others chromids such as pRetCFN42e from *R. etli* CFN42 [3]. However we have not yet tried to cure this replicon from the bacteria. In pRetCFN42e, Landeta *et al*. [49] analyzed a set of genes, most of which were also present in pRgrCCGE502b such as *hutUGHI* for histidine degradation; *pcaDCHGB* for protocatechuic acid degradation; *agpA*, *agaL1* and *agaL2*, involved in melobiose consumption; *nadABC* involved in the initial steps of NAD biosynthesis, *cls* responsible of cardiolipin synthesis, *thiMED* participating in the thiamine salvage pathway, *cobFGHIJKLM* involved in cobalamin biosynthesis (vitamin B12) and *cyoABCDE*, encoding the cytochrome O terminal oxidase. Additionally, on pRgrCCGE502b we found *minCDE* genes*,* involved in septum formation and *actP* for copper extrusion. Two essential genes required for growth in rich medium are present in pRetCFN42e, RHE_PE00001 and RHE_PE00024. *R. grahamii* showed an ortholog 68% identical to RHE_PE00001 also on pRgrCCGE502b, but RHE_PE00024 was not found in the genome. All these genes are present in single copy in each genome. Furthermore, some of the *R. phaseoli* Ch24-10 genes found to be highly expressed in maize or bean rhizosphere [1] were found to be conserved in pRgrCCGE502b (e.g. *cyoAB*, *hutUGH*, *apgA*, *cls*, *cobG* and *actP)*.

Most of the genes analyzed that were located on pRgrCCGE502b gave high identities, between 60 and 90%, to *Rhizobium* sp. CF122 and some with *R. mesoamericanum* STM625 gene sequences [21]. CF122 was isolated from *Populus deltoides* rhizosphere in North Carolina [15]. The ANI values we estimated for the genomes of *Rhizobium* sp. CF122 and *R. grahamii* or *R. mesoamericanum* were 87.5% and 87.8%, respectively. CF122 should correspond to a species other than *R. grahamii* or *R. mesoamericanum* considering its low ANI values with the reported related species.

ANI values between the megaplasmids in the “grahamii” group was nearly 85% (Figure 1B) but the percentage of conserved DNA between these replicons was around 14% (Figure 1C). ANI values of the corresponding chromosomes were estimated to be around 86% and conserved DNA around 75% (Figure 1B and C). In comparison with the *R. etli* CFN42 chromid, pRetCFN42e, these values were 83.28% and 13.75% (Additional file 2: Table S2).

### Symbiotic plasmid pRgrCCGE502a

Symbiosis genes were found on plasmid pRgrCCGE502a, most were located in a 108 kbp region. *nodABC* genes*,* responsible for synthesis of the Nod factor core, were located upstream of *nodSUIJHPQ*. NodS is an *N-*methyltransferase and NodU is a carbamoyltransferase responsible for adding substitutions at the C-2 and C-6 position, respectively, on the non-reducing *N-*acetyl-d-glucosamine of the Nod factor. *nodHPQ* gene products are involved in the sulfation of C-6 of the reducing terminus [50,51] and *NodIJ* are involved in the export of Nod factors [52,53]. The *R. grahamii* pSym also has *nodEF-hsnT.* NodE and NodF are involved in the synthesis of unsaturated fatty acids [54] and HsnT is an acyltransferase of non specified function. Based on the *nod* genes found, *R. grahamii* Nod factor structure was predicted as a chitin backbone of *N-*acetylglucosamine residues *N-*acylated with polyunsaturated fatty acids, *N-*methylated at the C-2 nonreducing terminal and carbamoylated at C-6 of the same residue. At the reducing end this Nod factor may be substituted at the C-6 position with sulfate.

**Figure 2:**
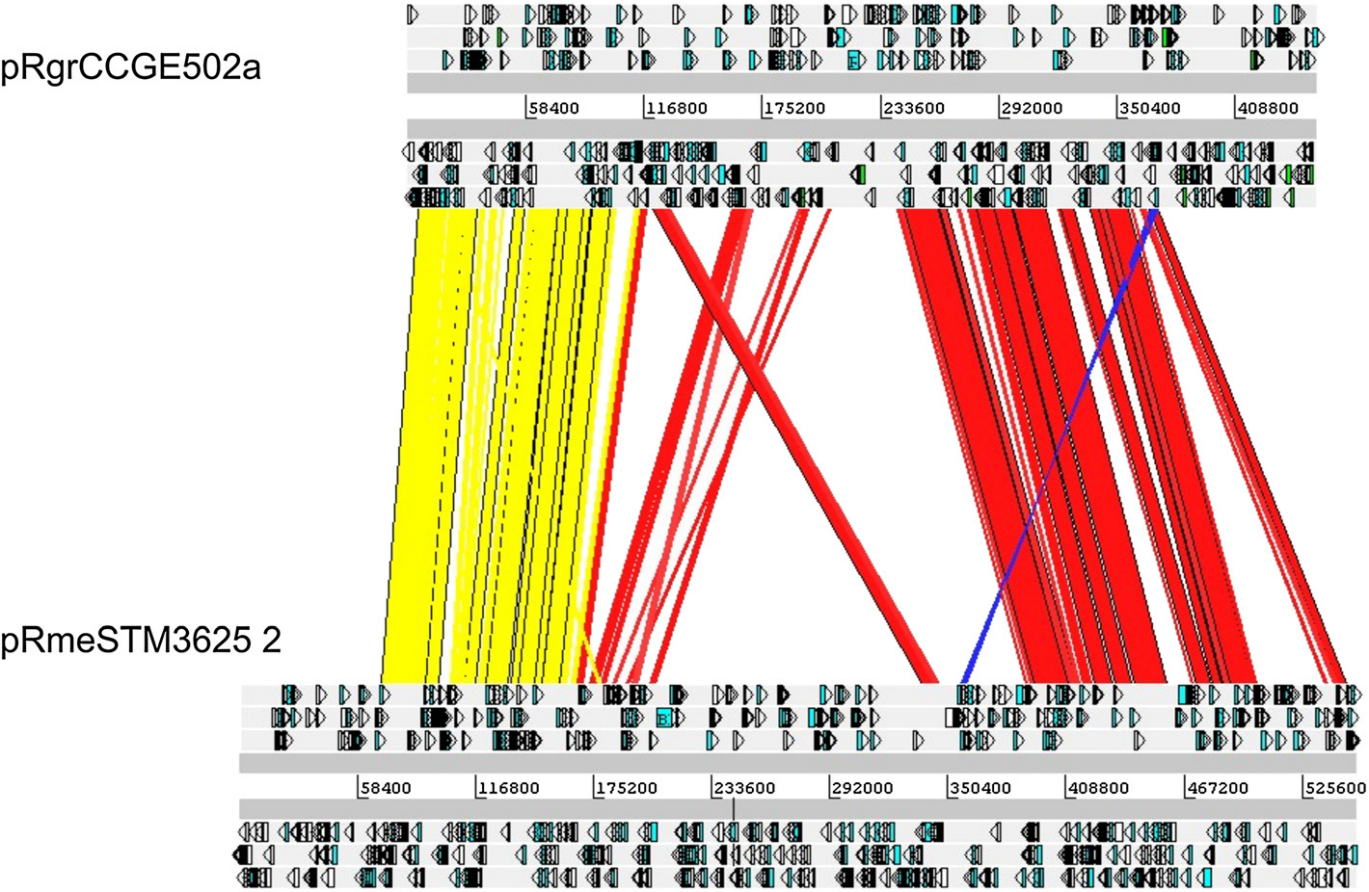
**Alignment of symbiotic plasmids of *R. grahamii *CCGE502 (pRgrCCGE502a) and *R. mesoamericanum *STM3625 (pRmeSTM3625 2).** Numbers indicate nucleotide positions and arrows the open reading frames in each replicon. Red and yellow lines indicate conserved regions with the same direction. Yellow lines show conserved symbiosis regions including *nif*, *fix* and *nod* genes. Blue lines indicate inverted conserved regions.

The symbiotic plasmids most similar to pRgrCCGE502a were those from *R. mesoamericanum* strains. A comparison of *nod* genes revealed that *R. grahamii* CCGE502 and *R. mesomericanum* STM3625 have almost the same nodulation gene products, ranging from 69% to 99% amino acid similarity (Figure 2). Despite this similarity, some differences were observed in overall pSym gene content as well as in individual *nod* genes (Figure 1C, Figure 2). *R. mesoamericanum* STM3625 lacks *nodEF-hsnT* but harbors two copies of *nodA* and three copies of *nodD*, while *R. grahamii* only presented one *nodA* and two *nodD* gene copies. *R. grahamii* had two *nodO* and one *nodM* gene copies located distant to the sym cluster. They encode a Ca-binding protein that is thought to form cation-specific channels in plant membranes [55] and a glucosamine 6-phosphate synthase, respectively. *R. mesoamericanum* STM3625 also has two *nodO* and one *nodM* gene copies; *nodO2* and *nodM* showed an identical genetic context, while *nodO1* is found in a different genetic context.

In relation to *nif/fix* genes, a complete set of genes for nitrogen fixation were found in *R. grahamii*. Some repeated genes, such as *nifQ* and *nifW* were also found*. nifW* had not been found in other *Rhizobium* species. There were two copies in both *R. grahamii* and *R. mesoamericanum* STM3625. Moreover, RGCCGE502_32751 (*nifW1*) had 92% similarity with BNN_260005 from *R. mesoamericanum* strain STM3625, and RGCCGE502_33006 (*nifW2*) had 98% similarity with BNN_270058 from *R. mesoamericanum* strain STM3625. *nifQ* was located next to *nifW* genes in *R. grahamii* and in *R. mesoamericanum* STM3625. *nifW* has an unknown function while *nifQ* is implicated in the processing of molybdenum, specifically for the biosynthesis of the iron-molybdenum cofactor of the nitrogenase. There are more *nif* genes in *R. grahamii* and *R. mesoamericanum* than in *E. meliloti* or *R. leguminosarum* sv. viciae (Table 2).

**Table 2:** ***nif *** **genes in *R. grahamii *** **CCGE502 and in other bacteria**

**Function**	**Gene**	**Kp**	**BTAi1**	**CFN42**	**CIAT 899**	**CCGE501**	**STM3625**	**CCGE502**	**Bd**	**Ml**	**Em**	**Rl 3841**
Regulation	*nifA*	**X**	**X**	**X**	**X**	**X**	**X**	**X**	**X**	**X**	**X**	**X**
FeMo-Co biosynthesis	*nifB*	**X**	**X**	**X**	**X**	**X**	**X**	**X**	**X**	**X**	**X**	**X**
Nitrogenase structural gene	*nifH*	**X**	**X**	**X**	**X**	**X**	**X**	**X**	**X**	**X**	**X**	**X**
Nitrogenase structural gene	*nifD*	**X**	**X**	**X**	**X**	**X**	**X**	**X**	**X**	**X**	**X**	**X**
Nitrogenase structural gene	*nifK*	**X**	**X**	**X**	**X**	**X**	**X**	**X**	**X**	**X**	**X**	**X**
FeMo-complex biosynthesis	*nifE*	**X**	**X**	**X**	**X**	**X**	**X**	**X**	**X**	**X**	**X**	**X**
FeMo-Co biosynthesis	*nifN*	**X**	**X**	**X**	**X**	**X**	**X**	**X**	**X**	**X**	**X**	**X**
Unknown function	*nifT*	**X**	**X**	**-**	**X**	**X**	**X**	**X**	**X**	**X**	**X**	**X**
FeMo-Co biosynthesis	*nifX*	**X**	**X**	**X**	**X**	**X**	**X**	**X**	**X**	**X**	**X**	
FeMo-Co biosynthesis	*nifQ*	**X**	**X**	**X**	**X**	**X**	**X**	**X**	**X**	**X**		
Unknown function	*nifW*	**X**	**X**	**X**	**X**	**X**	**X**	**X**	**X**	**X**		
Nitrogenase maturation	*nifZ*	**X**	**X**	**X**	**X**	**X**	**X**	**X**	**X**	**X**		
FeMo-Co biosynthesis	*nifS*	**X**	**X**	**X**	**X**	**X**	**X**	**X**	**X**	**X**		
FeMo-Co biosynthesis	*nifU*	**X**	**X**	**X**	**X**	**X**	**X**	**X**				
FeMo-Co biosynthesis	*nifV*	**X**	**X**									
Regulatory	*nifL*	**X**										
Electron donation	*nifF*	**X**										
Electron donation	*nifJ*	**X**										
FeMo-Co biosynthesis	*nifY*	**X**										
Nitrogenase maturation	*nifM*	**X**										

The comparison was done with *Klebsiella pneumoniae* as reference and other rhizobial strains with fully sequenced genomes. Kp, *Klebsiella pneumoniae*; BTAi1, *Bradyrhizobium* sp. BTAi1; CFN42, *R. etli* CFN42; CIAT899, *R. tropici* CIAT 899; CCGE501, *R. mesoamericanum* CCGE501; STM3625, *R. mesoamericanum* STM3625; CCGE502, *R. grahamii* CCGE502; Bd, *Bradyrhizobium diazoefficiens* USDA110; Ml, *Mesorhizobium loti* MAFF303099; Em, *Ensifer meliloti* 1021 and Rl 3841, *Rhizobium leguminosarum* sv. viciae 3841. In rhizobia, FixU functionally replaces NifT. Modified and updated from [56].

*R. grahamii* and *R. mesoamericanum* symbiotic plasmids showed an ANI of 94.54% (Table 3). Synteny analysis showed that the pSyms of both species are the most closely related (Figure 2), while only short and fragmented similarities were observed between the pSym of *R. grahamii* and those of *R. tropici* CIAT 899 and other species. In spite of the high sequence identity of genes between *R. grahamii* and *R. mesoamericanum*, the percentage of conserved DNA was only 42% to 51% (depending on the query sequence) of the total molecule (Table 3). In contrast, pSyms of phaseoli strains Ch24-10, CIAT652 and CFN42 showed higher conservation 88 to 95% (Table 3). Also, the percentage of conserved DNA was 96% among three symbiotic plasmids belonging to sv. tropici.

**Table 3:** Average nucleotide identity (ANI) and percentage of conserved DNA between symbiotic plasmids from different rhizobial strains

**Target**	**CCGE502**	**CCGE501**	**STM3625**	**CIAT 899**	***Rl *** **3841**	**CIAT652**	**CFN42**	**Ch24-10**
**Query**								
CCGE502		**94.54**	**94.45**	**87.62**	**83.07**	**87.13**	**87.03**	**87.18**
CCGE501	42.85		**98.07**	**88.1**	**81.83**	**87.03**	**86.66**	**86.99**
STM3625	39.58	61.44		**87.13**	**85.32**	**86.50**	**86.00**	**86.57**
CIAT 899	10.66	10.56	8.76		**82.42**	**86.21**	**86.24**	**86.19**
*Rl* 3841	1.52	1.01	2.39	1.45		**86.56**	**86.97**	**86.83**
CIAT652	6.91	5.95	6.21	3.69	2.09		**98.57**	**98.65**
CFN42	6.87	6.45	7.87	4.23	3.35	88.41		**98.83**
Ch24-10	6.03	6.18	5.79	3.33	2.34	90.62	82.97	

ANI values in bold numbers. Species and replicons compared: CCGE502, *R. grahamii* CCGE502 (pRgrCCGE502a); CCGE501, *R. mesoamericanum* CCGE501 (pRmeCCGE501c); STM3625, *R. mesoamericanum* STM3625 (pRmeSTM3625 2); CIAT 899, *R. tropici* CIAT 899 (pRtrCIAT899b); *Rl* 3841, *Rhizobium leguminosarum* sv. viciae 3841 (pRL10); CIAT652, *R. phaseoli* CIAT652 (pRphCIAT652b); CFN42, *R. etli* CFN42 (pRetCFN42d); Ch24-10, *R. phaseoli* Ch24-10 (pRphCh2410c).

### Phylogenetic analysis of RepB proteins of *R. grahamii* CCGE502

**Figure 3:**
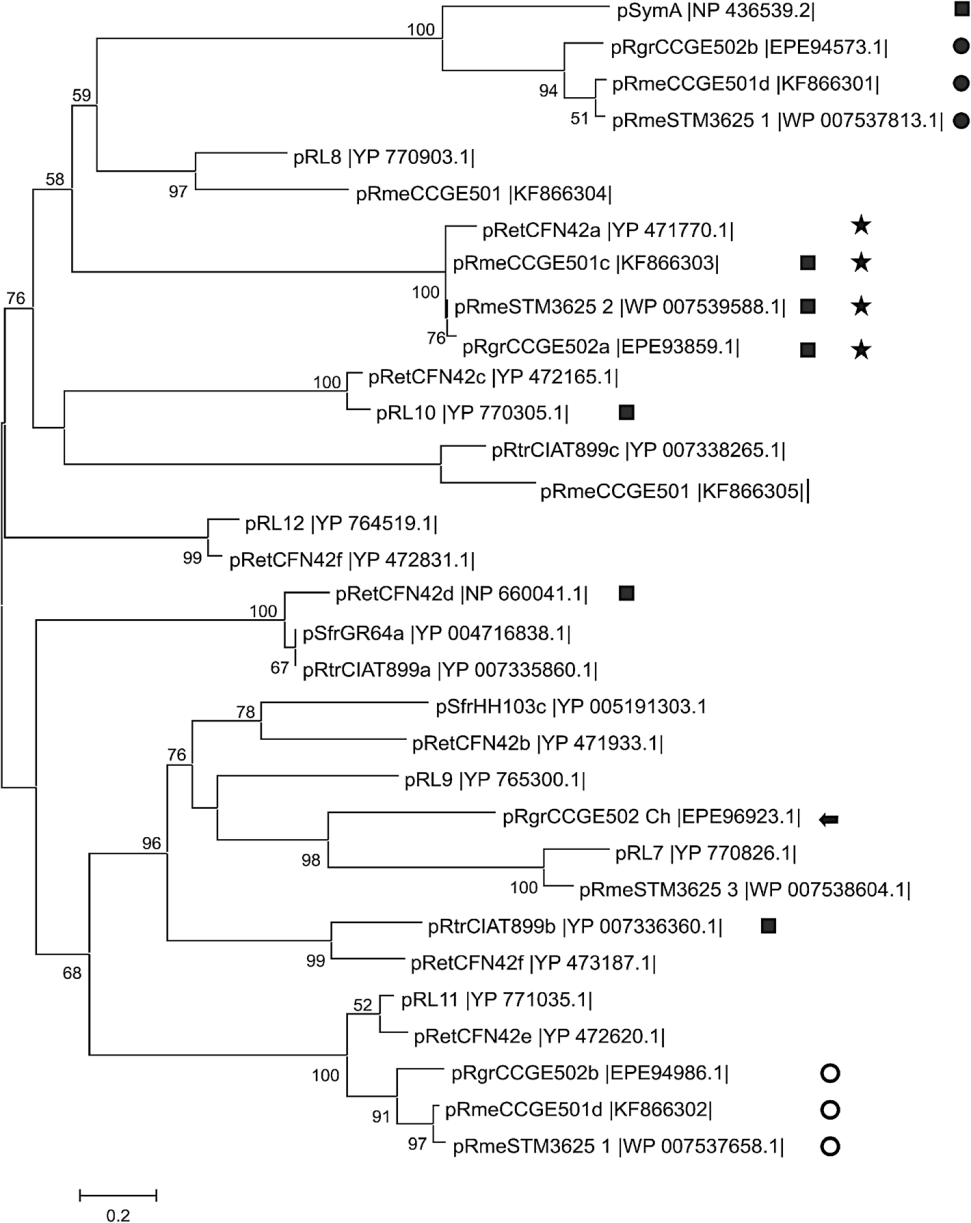
**Maximum likelihood phylogeny of RepB proteins.** LG + I + G + F was used as model of amino acid substitution. Labels indicate the replicon and the GenBank accession numbers. Squares indicate proteins with genes found in symbiotic plasmids, circles indicate RepB of *R. grahamii* and *R. mesoamericanum* megaplasmids: filled circles specify proteins encoded by genes organized in a *repABC* operon and empty circles specify RepB proteins encoded in a *repAB* operon. Stars indicate proteins of *R. grahamii* and *R. mesoamericanum* encoded in symbiotic plasmids, together with RepB of pRetCFN42a. The arrow indicates the chromosomal RepB. Numbers close to tree nodes indicate branch support evaluated by the Shimodaira–Hasegawa-like approximate likelihood-ratio test (only values higher than 50% are shown). Scale bar, 0.2 amino acid substitutions per site.

Rhizobial plasmids have *repABC* operons involved in their replication and maintenance. RepA and RepB are proteins that participate in active plasmid segregation and RepC is the replication initiator protein [57]. Additional *repC* gene copies have been found separated from *repAB* and may have different evolutionary origins [58]. pRgrCCGE502a has one independent *repC* gene copy located at the nodulation cluster. Four *repB* gene copies were found, one encoded in the genomic island of CCGE502 chromosome, two in pRgrCCGE502b and one in pRgrCCGE502a (Figure 3). Megaplasmid RepB proteins from *R. grahamii* and *R. mesoamericanum* were closely related (Figure 3, filled and empty circles) as well as those of the symbiotic plasmids respectively (Figure 3, stars). RepB of *R. etli* pRetCFN42a (YP_471770.1) was related to the corresponding sequences from the symbiotic plasmids in the “grahamii” group (Figure 3, stars). In the symbiotic plasmids, *repABC* operons were located next to Mating Pair Formation (Mpf) and DNA transfer and replication (Dtr) system genes.

The presence of a *repB* gene localized in the chromosome may be considered as further evidence that this region originated from a plasmid (Figure 3, arrow). It grouped with the corresponding genes from pRL7 of *R. leguminosarum* sv. viciae and from pRmeSTM3625 3 of *R. mesoamericanum* STM3625. A phylogenetic analysis of RepC proteins revealed similar results (not shown) to those obtained with RepB phylogenies except that the chromosomal RepC protein grouped with the corresponding protein from pRetCFN42d of *R. etli*.

### Conjugative transfer of the symbiotic plasmid and megaplasmid of *R. grahamii* CCGE502

**Figure 4:**
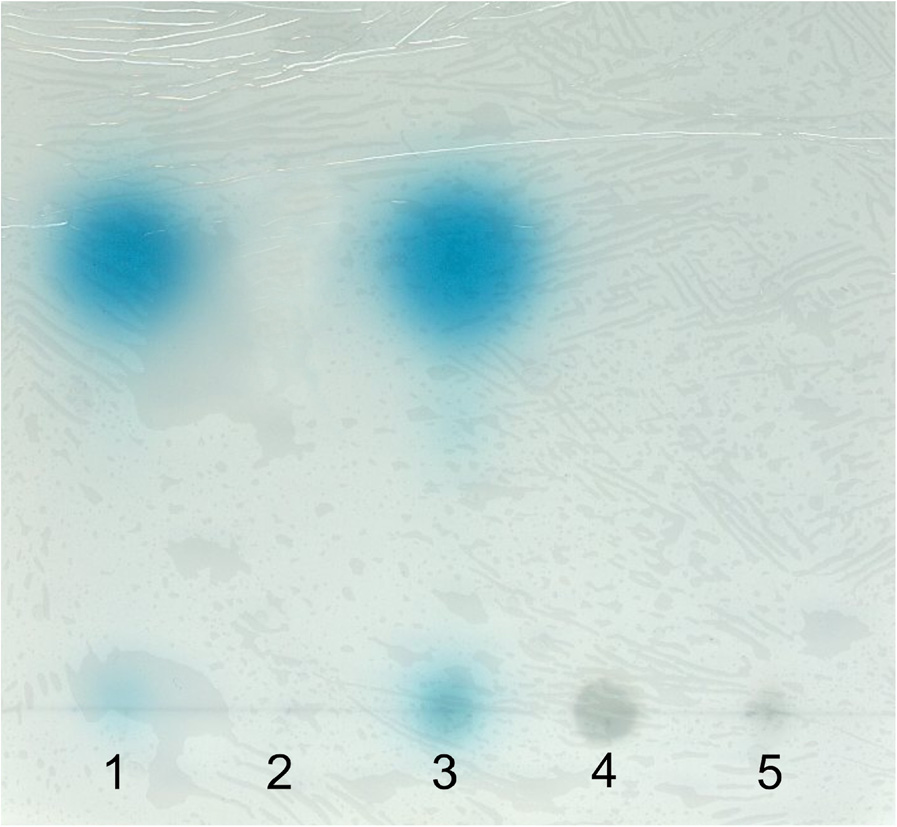
**Thin-layer chromatogram of the AHLs produced by *R. grahamii *CCGE502 and derivatives.** 1) *R. grahamii* CCGE502 wild type strain; 2) *R. grahamii* CCGE502a*ΔtraI;* 3) *A. tumefaciens* GMI9023 (pRgrCCGE502a: GFP); 4) *A. tumefaciens* GMI9023 (pRgrCCGE502a*ΔtraI*) and 5) *A. tumefaciens* GMI9023 (negative control). Equal amounts of sample were loaded in each lane, except at lane 3 where the sample was ten-fold concentrated.

The organization of the *trb* cluster (Mpf proteins) and *tra* cluster (Dtr proteins) is identical in *R. grahamii* CCGE502 and *R. etli* CFN42 (identities of 95%), only differing in that *cinR* is present in pRetCFN42a but absent in the symbiotic plasmid pRgrCCGE502a. The high similarity among the conjugative transfer genes could suggest a similar regulation of plasmid transfer. In *R. etli* CFN42, three genes present in pRetCFN42a are necessary for plasmid transfer dependent on quorum sensing: *traI*, *N-*acyl-homoserine synthase, *cinR* and *traR,* both encoding transcriptional regulators [25]. Notably, mobilization of pRetCFN42d (pSym) depends on its cointegration with pRetCFN42a [59]. *R. grahamii* CCGE502 has *traI* (RGCCGE502_33766) and *traR* (RGCCGE502_33821) genes in the symbiotic plasmid. A *traI* mutant of *R. grahamii,* CCGE502a*ΔtraI* did not produce AHLs (Figure 4). As Figure 4 shows, an *A. tumefaciens* GMI9023 transconjugant carrying pRgrCCGE502a:GFP produced all AHLs present in *R. grahamii*, albeit at a highly reduced level (see below), suggesting that RGCCGE502_33766 is responsible for all the spots detected by TLC.

The symbiotic plasmid of *R. grahamii* CCGE502a:GFP could be transferred at a frequency of *ca.* 10^-6^ transconjugants per donor cell to the plasmid-free *A. tumefaciens* GMI9023 strain [28], but this transfer was abolished when the *traI*-mutant was assessed (fewer than 3.0 × 10^-1^ transconjugants per donor cell). Thus, we considered that conjugative transfer of pRgrCCGE502a was regulated by quorum sensing as occurs with pRetCFN42a. Although pRgrCCGE502a could be transferred to *A. tumefaciens* GMI9023, transfer of this pSym to *R. mesoamericanum* CCGE501, *R. etli* CFN2001 [25], *Sinorhizobium fredii* GR64-4 [26], *Ensifer meliloti* SmA818R [27], *R. phaseoli* Ch24-10, *Rhizobium* sp. LPU83 [27] and *R. endophyticum* CCGE2052 [11] was tried unsuccessfully.

Due to the close relationship of RepC proteins of pRgrCCGE502a and pRetCFN42a (RGCCGE502_33751 and RHE_PA00182), we considered that they could be incompatible. Nevertheless a plasmid cured strain (without pRetCFN42a and pRetCFN42d) also was unable to act as a recipient. Furthermore, pRgrCCGE502a:GFP could not be mobilized from the *A. tumefaciens* transconjugants. Remobilization experiments were done either from GMI9023 (pRgrCCGE502a:GFP) or GMI9023 (pRgrCCGE502a:GFP, pRgrCCGE502b:Km) to another GMI9023 (Sp^R^) and no transconjugants were obtained. The production of AHLs in the genomic background of *A. tumefaciens* is at least ten-fold lower than in *R. grahamii* (Figure 4) and this event may explain why pRgrCCGE502a:GFP could not be transferred from GMI9023. However *A. tumefaciens* overexpressing the AHLs of *R. grahamii,* GMI9023 (pRgrCCGE502a:GFP, pBBR1MCS2*::traI*) was not able to mobilize the symbiotic plasmid, indicating that additional factors are needed. Some of these factors could be encoded in the chromosome and thus they are not present when transfer is assayed from *A. tumefaciens* carrying the plasmids of *R. grahamii* as donor.

**Figure 5:**
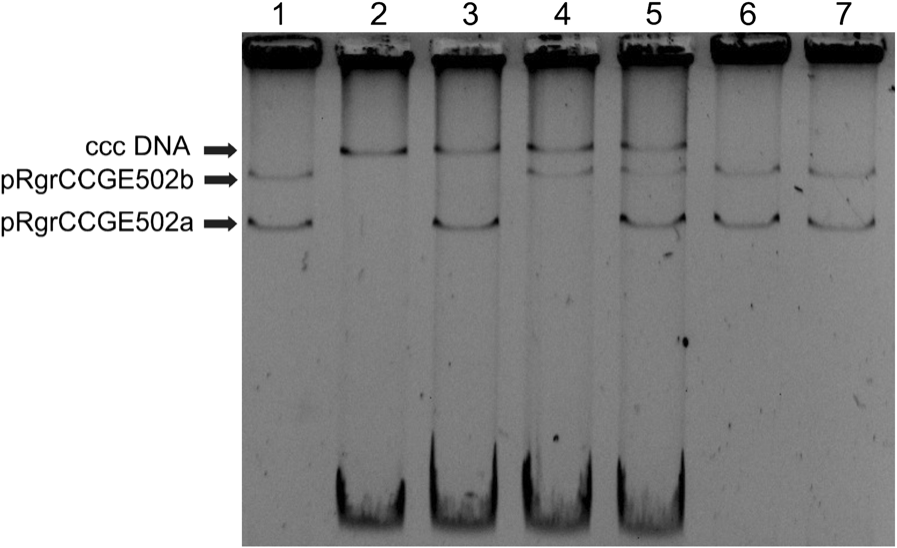
**Plasmid profiles in Eckhardt gels.** 1) *R. grahamii* CCGE502, 2) *A. tumefaciens* GMI9023, 3) *A. tumefaciens* GMI9023 (pRgrCCGE502a: GFP), 4) *A. tumefaciens* GMI9023 (pRgrCCGE502b:Km), 5) *A. tumefaciens* GMI9023 (pRgrCCGE502a: GFP, pRgrCCGE502b:Km), 6) *R. grahamii* CCGE502a: GFP and 7) *R. grahamii* CCGE502b:Km. Ccc DNA: closed circular chromosome of *A. tumefaciens* GMI9023.

By triparental conjugation (using pRK2013 as helper) megaplasmid pRgrCCGE502b:Km was transferred to *A. tumefaciens* GMI9023 or GMI9023 (pRgrCCGE502a:GFP) but it could not be transferred to *Rhizobium* species such as *R. etli* CFN42*.* Figure 5 shows the plasmid profile of *R. grahamii* wild type strain and *A. tumefaciens* GMI9023 carrying pRgrCCGE502a or pRgrCCGE502b or both plasmids.

## Discussion and conclusions

When comparing genomes from closely related rhizobial species (e.g. *R. tropici* and *R. rhizogenes* or *R. leguminosarum* and *R. etli*), it was observed that there is a larger degree of conservation in the chromosomes than in the ERs [3,60]. We confirmed here a high degree of conservation between the chromosomes of strains in the “grahamii” group, namely *R. grahamii* CCGE502, *R. mesoamericanum* CCGE501 and STM3625, as well as *Rhizobium* sp. CF122. However, in other cases a larger degree of nucleotide conservation has been observed in the symbiotic plasmids (e.g. symbiotic plasmids from the tropici or phaseoli symbiovars) than in chromosomes. In *R. grahamii* and *R. mesoamericanum* we observed the largest nucleotide identity in pSyms (ANI around 94%), but not as large as among tropici and phaseoli symbiotic plasmids with ANI of 99 or 98% respectively (Table 3). The conservation of pSyms may be explained by the lateral transfer of a successful plasmid (epidemic plasmid in terms of Souza *et al.*[61]) or a wandering plasmid among different rhizobial lineages [62] or from being a recently evolved replicon. In the case of the phaseoli plasmids we favored the latter explanation [4,62-64]. Anyhow, it seems reasonable to consider that limited replicon transfer among related species would lead to an isolated evolutionary history linked to a single genomic background. The phaseoli and tropici plasmids have been found to be conjugative with a high frequency of transfer among rhizobia [65], and the “phaseoli” pSym is found in distantly related species such as *R. giardinii* or *R. gallicum*[66]. In contrast we were unable to transfer *R. grahamii* ERs to other rhizobia. It is worth noting that tropici symbiotic plasmids are more conserved than phaseoli ones, and both are more conserved than the grahamii group pSyms. It is tempting to suggest that genome conservation among distinct species is related to transferability. On the other hand, transfer of plasmids to novel hosts can also detonate their evolution by picking up new genetic information (that would affect the genomic content) from other genomic backgrounds. We do not know if in natural habitats or in the presence of a microbial community, the lack of transferability of *R. grahamii* ERs holds true. Besides, the limited conservation of pSyms among *R. grahamii* and *R. mesoamericanum* suggests that they are not frequently interchanged among these species. Transfer of the *R. grahamii* symbiotic plasmid to *Agrobacterium* was dependent on quorum sensing, a mechanism that regulates transfer of plasmids in rhizobia [25,67] and agrobacteria [68,69]. This lack of ER flow and existence of a genetic barrier could be due to different mechanisms, such as DNA restriction/methylation systems or to surface or entry exclusion systems. Surface exclusion at the level of formation of stable mating aggregates and entry exclusion seem to inhibit conjugation in a later step of the mating aggregate [70,71]. Limited transfer may be due to a system similar to CRISPR/Cas, an adaptive immunity system found in Archaea and bacteria that eliminates virus or plasmids in a new host [72,73]. These possibilities deserve further research.

Putative chromids (megaplasmids) in the grahamii group have a lower percentage of gene content conservation than the chromosomes and symbiotic plasmids, in spite of their fairly high ANI values (Figure 1B and C). Considering the conserved genomic content in chromosomes, symbiotic plasmids and putative chromids in the grahamii group, there clearly are three different degrees of conservation (Figure 1C). We suggest a layout where the rhizobial genome is a 3 gear genome with different rates of change in each of the replicon types. In animals and plants, different regions of the genome exhibit variable levels of genetic divergence between populations (reviewed in Nosil *et al.*[74]).

The extrachromosomal replicons of *R. grahamii* CCGE502 were related to those from *R. mesoamericanum.* An exception is the plasmid integrated in the *R. grahamii* chromosome for which no equivalent plasmid was found in *R. mesoamericanum* or in other rhizobia. However some common genes were found in the *R. grahamii* integrated replicon and in other *Rhizobium* species. ER organization plasticity was reported previously in rhizobia with the integration of plasmids or megaplasmids into the chromosome [75,76]. This seems to have occurred in *R. grahamii* CCGE502 as we report here.

It is noteworthy that some of the genes highly expressed in *R. phaseoli* Ch24-10 when colonizing roots were found to be conserved in *R. grahamii* CCGE502 and do not seem to constitute a single genomic island, instead they were patchily distributed in pRgrCCGE502b. Such genes may have an important role in root colonization and seem to have been preserved during rhizobial divergence.

### Availability of supporting data

The data set supporting the results of this article is available in the Treebase repository, http://treebase.org/treebase-web/search/study/summary.html?id=14994.

## Competing interests

The authors declare that they have no competing interests.

## Authors’ contributions

MJA obtained the bacterial DNA and together with LL assembled and worked on the genome. Also, MJA carried out the molecular genetics experiments and wrote the manuscript. MAR assisted in laboratory experiments. EOO participated in sequence annotation, analysis and prepared some illustrations. GTT participated in design and discussion of genetics experiments. JM and coworkers performed plasmid profiles, isolated a novel *R. grahamii* strain, helped closing gaps and participated in discussion. EMR conceived the study, wrote and revised the manuscript. All authors approved the final manuscript.

## Supplementary Material

Additional file 1: Table S1 Average nucleotide identity (ANI) and percentage of conserved DNA between chromosomes. Click here for file

Additional file 2: Table S2 Average nucleotide identity (ANI) and percentage of conserved DNA between chromids. Click here for file
