# Supplementary material for: Characterization of Rhizobium grahamii extrachromosomal replicons and their transfer among rhizobia
Source: BMC Microbiol. 2014 Jan 8;14:6. doi: 10.1186/1471-2180-14-6 (PMC3898782; doi:10.1186/1471-2180-14-6)
Supplement: Additional file 1: Table S1 — Average nucleotide identity (ANI) and percentage of conserved DNA between chromosomes. [file 1471-2180-14-6-S1.docx]

| **Target**  **Query** | CCGE502 | CCGE501 | SMT3625 | CIAT 899 | *Rl* 3841 | CIAT652 | CFN42 | Ch24-10 |
| --- | --- | --- | --- | --- | --- | --- | --- | --- |
| CCGE502 |  | **86.96** | **86.95** | **84.46** | **84.35** | **84.35** | **84.35** | **84.36** |
| CCGE501 | 73.73 |  | **96.97** | **84.44** | **84.29** | **84.29** | **84.17** | **84.25** |
| SMT3625 | 74.79 | **96.97** |  | **84.55** | **84.31** | **84.30** | **84.17** | **84.26** |
| CIAT 899 | 18.44 | 15.27 | 14.76 |  | **84.57** | **84.71** | **84.51** | **84.66** |
| *Rl* 3841 | 26.37 | 22.08 | 22.21 | 20.39 |  | **88.53** | **88.12** | **88.48** |
| CIAT652 | 29.46 | 24.15 | 24.22 | 22.3 | 75.62 |  | **89.92** | **98.35** |
| CFN42 | 27.1 | 23.64 | 23.68 | 21.6 | 76.63 | 82.74 |  | **89.93** |
| Ch24-10 | 29.26 | 24.36 | 24.10 | 22.33 | 75.78 | 93.62 | 80.90 |  |

**Table S1.** Average nucleotide identity (ANI) (bold numbers) and percentage of conserved DNA between chromosomes. In the columns sequences used as reference. Chromosomes compared were those of CCGE502, *R. grahamii* CCGE502; CCGE501, *R. mesoamericanum* CCGE501; STM3625, *R. mesoamericanum* STM3625; CIAT899, *R. tropici* CIAT899; *Rl* 3841, *Rhizobium leguminosarum* sv. viciae 3841; CIAT652, *R. phaseoli* CIAT652; CFN42, *R. etli* CFN42; Ch24-10, *R. phaseoli* Ch24-10.
