# Supplementary material for: Characterization of Rhizobium grahamii extrachromosomal replicons and their transfer among rhizobia
Source: BMC Microbiol. 2014 Jan 8;14:6. doi: 10.1186/1471-2180-14-6 (PMC3898782; doi:10.1186/1471-2180-14-6)
Supplement: Additional file 2: Table S2 — Average nucleotide identity (ANI) and percentage of conserved DNA between chromids. [file 1471-2180-14-6-S2.docx]

| **Target**  **Query** | pRgrCCGE502b | pRmeCCGE501d | pRmeSTM3625 1 | pRtrCIAT899c | pRL11 | pRL12 | pRphCIAT652a | pRphCIAT652c | pRetCFN42e | pRphCh24-10d |
| --- | --- | --- | --- | --- | --- | --- | --- | --- | --- | --- |
| pRgrCCGE502 |  | **84.68** | **84.82** | **82.94** | **83.34** | **82.95** | **83.45** | **84.18** | **83.28** | **84.06** |
| pRmeCCGE501 | 14.55 |  | **95.14** | **83.58** | **83.15** | **85.84** | **82.99** | **86.54** | **84.02** | **87.33** |
| pRmeSTM3625 1 | 13.63 | 74.6 |  | **85.24** | **83.03** | **86.03** | **83.21** | **85.30** | **84.30** | **86.50** |
| pRtrCIAT899c | 1.40 | 2.39 | 2.98 |  | **83.83** | **83.20** | **83.57** | **2.99** | **83.52** | **83.81** |
| pRL11 | 8.72 | 8.97 | 8.91 | 10.55 |  | - | **88.48** | **87.85** | **88.20** | **90.29** |
| pRL12 | 2.04 | 4.36 | 4.73 | 6.34 | - |  | **89.92** | **86.43** | **88.44** | **86.58** |
| pRphCIAT652a | 17.11 | 13.41 | 15.92 | 16.7 | 67.48 | 0.25 |  | - | **91.13** | **0.06** |
| pRphCIAT652c | 2.11 | 4.87 | 4.76 | 5.7 | 0.07 | 30.03 | - |  | **87.32** | **97.64** |
| pRetCFN42e | 13.75 | 16.08 | 15.33 | 15.47 | 62.37 | 0.32 | 65.51 | 3.20 |  | **87.58** |
| pRphCh24-10d | 2.33 | 6.52 | 7.06 | 5.86 | 0.52 | 27.60 | 0.02 | 75.91 | 1.66 |  |

**Table S2.** Average nucleotide identity (ANI) (bold numbers) and percentage of conserved DNA between chromids. In the columns sequences used as reference. Species and replicons compared were: *R. grahamii* CCGE502 (pRgrCCGE502b); *R. mesoamericanum* CCGE501 (pRmeCCGE501d); *R. mesoamericanum* STM3625 (pRmeSTM3625 1); *R. tropici* CIAT 899 (pRtrCIAT899c); *R. leguminosarum* sv. viciae 3841 pRL11 and pRL12; *R. phaseoli* CIAT652 pRphCIAT652a and pRphCIAT652c, *R. etli* CFN42 (pRetCFN42e); *R. phaseoli* Ch24-10 (pRphCh2410d).
